# SFRP1 decreases WNT-Mediated M2 macrophage marker expression in breast tissue

**DOI:** 10.1007/s00262-024-03638-0

**Published:** 2024-03-30

**Authors:** Kelly J. Gregory, Holly Mason, Jesse Casaubon, Sallie S. Schneider

**Affiliations:** 1 Pioneer Valley Life Sciences Institute, Baystate Medical Center, Springfield, MA 01199 USA; 2 Biology Department, University of Massachusetts, Amherst, MA 01003 USA; 3 Department of Surgery, UMass Chan Medical School- Baystate Medical Center, Springfield, MA 01107 USA; 4 Veterinary and Animal Sciences, University of Massachusetts, Amherst, MA 01003 USA

**Keywords:** Macrophage polarization, Breast cancer

## Abstract

The Wnt family of secreted proteins are involved in mammary gland development and tumorigenesis. It has recently been shown that Wnt ligands promote M2 macrophage polarization and so we sought to determine the effects of a Wnt signaling antagonist, Secreted Frizzled Related Protein 1 (SFRP1), on M2 marker expression. We measured a murine M2 marker (*Arg1*) in mice with a targeted deletion of *Sfrp*1 during different stages of mammary gland development including puberty, pregnancy, and lactation, as well as in response to obesity. Next, to determine whether Wnt signaling/antagonism affects human M2 markers (*CD209* and *CCL18*), we treated a human patient derived explant (PDE) breast tissue sample with exogenous Wnt3a in the presence and absence of rSFRP1. Finally, we expanded our PDE study to 13 patients and performed bulk RNAseq analysis following the treatment described above. We found that in loss of *Sfrp*1 in the murine mammary gland increased *Arg*1 expression. Moreover, we showed that Wnt3a increases *CD209* and *CCL18* mRNA and protein expression in breast PDEs and that their expression is decreased in response to rSFRP1. Our RNAseq analysis unveiled novel genes that were affected by Wnt3a treatment and subsequently reversed when rSFRP1 was added. Validation of these data exhibited that chemokines involved in promoting macrophage polarization and cancer metastasis, including *CCL11* and *CCL26*, were stimulated by Wnt3a signaling and their expression was abrogated by treatment with rSFRP1. Our data suggest that SFRP1 may be an important mediator that tempers Wnt signaling in the tumor microenvironment.

**Supplementary Information:**

The online version contains supplementary material available at 10.1007/s00262-024-03638-0.

## Introduction

The Wnt family of growth factors are secreted proteins largely known for their involvement in cell fate and migration during development as well as in tissues with high rates of stem cell activity and turnover [1, 2]. The best characterized Wnt pathway is the canonical Wnt/β-catenin pathway whereby Wnt ligands bind to receptors comprised of Frizzled proteins leading to the stabilization of β-catenin and activation of β-catenin-responsive gene expression. Secreted frizzled-related proteins (SFRPs) are a family of Wnt antagonists that can antagonize Wnt signaling by binding to Wnt ligands and thus prevent ligand-receptor interactions and signal transduction [3].

SFRP1 is believed to be a tumor suppressor in human breast cancer. Hypermethylation and loss of expression of SFRP1 is frequently associated with pre-malignant lesions in breast tissue [4–7]. Moreover, loss of SFRP1 expression is associated with poor overall survival in patients with breast cancer [8]. While most studies have examined SFRP1 as an autocrine tumor suppressor, more recent data has suggested a more complex mechanism which could involve cross talk with the tumor microenvironment (TME). Of particular interest to this study, when *Sfrp1*^*−/−*^ mice were challenged with a high fat diet, there was an augmented immune response which included an increase in the number of macrophages in the mammary gland [9].

Wnt ligands have been demonstrated to directly promote murine macrophage polarization and activity [10, 11]. Macrophages comprise a subset of immune cells that are phagocytic in nature and are present in almost all tissues. Depending on the type of signal in the microenvironment, macrophages are polarized into distinct phenotypes. Classically activated M1 macrophages are involved in Th1 responses to pathogens and play an important role in both innate host defenses and tumoricidal activities by expressing pro-inflammatory cytokines and are therefore considered as antitumor macrophages [12]. Alternatively activated M2 macrophages are induced by Th2 cytokines and are generally divided into 4 subtypes critical for wound healing (M2a), immunoregulation (M2b) immunosuppression (M2c) and tumor development (M2-like/M2d) [13]. Tumor-associated macrophages (TAMs) are more frequently observed to be of the M2 type and secrete soluble factors which typically facilitate angiogenesis, tumor initiation, growth, and metastasis [14].

In this study we sought to determine whether the deletion of *Sfrp1* is associated with higher levels of markers consistent with M2 polarized macrophages during developmental timepoints associated with Wnt activity. Furthermore, we extended this to human tissues with the use of patient derived benign breast tissue explants. We examined markers associated with M2 polarization following exposure to Wnt3a with or without rSFRP1 by immunohistochemistry and performed RNAseq to gauge the impact of Wnt exposure on the transcriptome.

## Methods

### Mice

All procedures were performed in accordance with the NIH guidelines for the ethical treatment of animals and were approved by the Baystate Medical Center Institutional Animal Care and Use Committee before initiating these studies. Female 129/C57Blk6 mice (*n* = 6) were individually housed in plastic cages with food and water provided continuously and maintained on a 12:12 light cycle. Mice (*n* = 10/genotype) were placed on a high fat diet [(HFD) Bio-Serv (#F1850) containing 36.0% fat, 36.2% carbohydrate, and 20.5% protein] starting at 10 weeks of age for 12 weeks.

### Patient derived explants

Fresh breast tissue was obtained from 13 women undergoing breast surgery at Baystate Medical Center, Springfield, MA who are enrolled in the Rays of Hope Center for Breast Research Registry (IRB Baystate Health, Springfield, MA protocol Number 568088; Table 1). The tissue was cultured on Surgifoam as previously described [15–18]. The media was supplemented with 250 ng/mL recombinant human Wnt3a (R&D systems, Minneapolis, MN) and ± 1 μg/mL recombinant human SFRP1 (Sigma-Aldrich, Burbank, CA). Following a 72-h incubation, half of the tissue and media was flash frozen at −80 °C for RNA isolation and the other half of the tissue was formalin fixed and paraffin embedded for immunohistochemistry. The conditioned media was also collected for ELISA assays.

**Table 1:** Demographic information for breast patient derived explants

SID #	Age	BMI	Surgerical procedure	Race/ethnicity
1281	40	35.1	Bilateral Mastectomy	Latina-African American
1288	55	31.8	Single Mastectomy	Latina
1296	63	21.7	Bilateral Mastectomy	White-Ashkenazi
1297	61	37.5	oncoplastic reduction	White
1304	66	23.3	Single Mastectomy	White-Ashkenazi
1306	49	48.8	Oncoplastic Reduction	White
1307	53	32.7	Bilateral Mastectomy	white-FC
1310	54	35.5	Single Mastectomy	African American
1322	41	28.8	Bilateral Mastectomy	White-Ashkenazi
1395	19	30.7	Reduction	White
1397	72	21.2	Bilateral Mastectomy	White
1407	46	32	Single Mastectomy	White-Dutch
1408	74	26.9	Single Mastectomy	White-French Canadian
1412	46	31.4	Single Mastectomy	Latina

### RNA isolation and real-time PCR

Total RNA was extracted from the sixth inguinal mammary glands of 10-week-old, pregnant day 8, and lactating day 6 animals using an acid-phenol extraction procedure, according to the manufacturer’s instructions (Trizol, Invitrogen, Carlsbad, CA). Total RNA was harvested from PDEs as described above with an additional purification step using the Direct-zol RNA mini RNA kit (Zymo Research, Irvine, CA) according to the manufacturer’s instructions. Relative levels of the mRNA expression of target genes were determined by quantitative real-time PCR using the QuantStudio™ 3 real-time PCR system (Thermo Fisher Scientific). The PCR primer sequences for *Actb*, *ACTB*, *CD209*, and *CCL18* have been described [17, 19, 20] and primers shown in Supplementary Table S1 were designed to cross exon junctions using Primer BLAST (National Center for Biotechnology information, NCBI; https://www.ncbi.nlm.nih.gov/ tools/primer‐blast/). The assays were performed using the 1-Step Brilliant^®^ SYBRIII^®^ Green QRT-PCR Master Mix Kit (Agilent) as described previously [21]

### Immunohistochemistry

Immunohistochemistry (IHC) was performed on a DakoCytomation autostainer as previously described [17] using the primary antibody CD209 (Abcam 1:500; ab218419) for 30 min. Immunoreactivity was visualized by incubation with chromogen diaminobenzidine (DAB) for 5 min. Images were captured with an Olympus BX41 light microscope using SPOT Software 5.1 (SPOT^™^Imaging Solutions, Detroit, MI).

### Enzyme linked immunosorbent assay

Supernatant was collected from PDE cultures for the analysis of CCL18 protein secretion using the PerkinElmer AlphaLISA Detection kit (AL3106C/F) according to the manufacturer’s instructions using an EnSight Multimode plate reader (PerkinElmer, Waltham, MA).

### Library construction, quality control and sequencing

Messenger RNA was purified from total RNA using poly-T oligo-attached magnetic beads. After fragmentation, the first strand cDNA was synthesized using random hexamer primers, followed by the second strand cDNA synthesis using either dUTP for directional library or dTTP for non-directional libraryQuantified libraries were pooled and sequenced on Illumina platforms (Illumina Inc., San Diego, CA) according to effective library concentration and data amount.

### Clustering and sequencing

The clustering of the index-coded samples was performed according to the manufacturer’s instructions. After cluster generation, the library preparations were sequenced on an Illumina platform and paired-end reads were generated.

### Data analysis

For quality control, raw data (raw reads) of fastq format were firstly processed through in-house perl scripts. In this step, clean data (clean reads) were obtained by removing reads containing adapter, reads containing ploy-N and low quality reads from raw data. At the same time, Q20, Q30, and GC content the clean data were calculated. All the downstream analyses were based on the clean data with high quality.

### Quantification of gene expression level

Feature Counts v1.5.0-p3 was used to count the reads numbers mapped to each gene. And then FPKM of each gene was calculated based on the length of the gene and reads count mapped to this gene. FPKM, expected number of Fragments Per Kilobase of transcript sequence per Millions base pairs sequenced, considers the effect of sequencing depth and gene length for the reads count at the same time, and is currently the most commonly used method for estimating gene expression levels.

### Enrichment analysis of differentially expressed genes

Gene Ontology (GO) enrichment analysis of differentially expressed genes was implemented by the cluster Profiler R package, in which gene length bias was corrected. GO terms with corrected *P* value less than 0.05 were considered significantly enriched by differential expressed genes. KEGG is a database resource for understanding high-level functions and 3 utilities of the biological system, such as the cell, the organism and the ecosystem, from molecular-level information, especially large-scale molecular datasets generated by genome sequencing and other high-through put experimental technologies (http://www.genome.jp/kegg/). We used cluster Profiler R package to test the statistical enrichment of differential expression genes in KEGG pathways. The Reactome database brings together the various reactions and biological pathways of human model species. Reactome pathways with corrected *P* value less than 0.05 were considered significantly enriched by differential expressed genes. The DO (Disease Ontology) database describes the function of human genes and diseases. DO pathways with corrected *P* value less than 0.05were considered significantly enriched by differential expressed genes. The DisGeNET database integrates human disease-related genes. DisGeNET pathways with corrected *P* value less than 0.05 were considered significantly enriched by differential expressed genes. We used cluster Profiler software to test the statistical enrichment of differentially expressed genes in the Reactome pathway, the DO pathway, and the DisGeNET pathway.

### Gene set enrichment analysis

Gene Set Enrichment Analysis (GSEA) is a computational approach to determine if a pre-defined Gene Set can show a significant consistent difference between two biological states. The genes were ranked according to the degree of differential expression in the two samples, and then the predefined Gene Set were tested to see if they were enriched at the top or bottom of the list. Gene set enrichment analysis can include subtle expression changes. We use the local version of the GSEA analysis tool http://www.broadinstitute.org/gsea/index.jsp, GO, KEGG, Reactome, DO, and DisGeNET data sets were used for GSEA independently.

### Statistical analysis

Group means were compared using Student’s *t*-tests (Graphpad Prism) and results with *P* < 0.05 were considered significant. A test for outliers was performed on all data sets using a Grubbs’ test (GraphPad QuickCalcs) and statistical outliers were not included data analysis.

## Results

### SFRP1 deletion results in higher levels of M2 markers in mouse mammary gland during periods of critical development

**Fig. 1:**
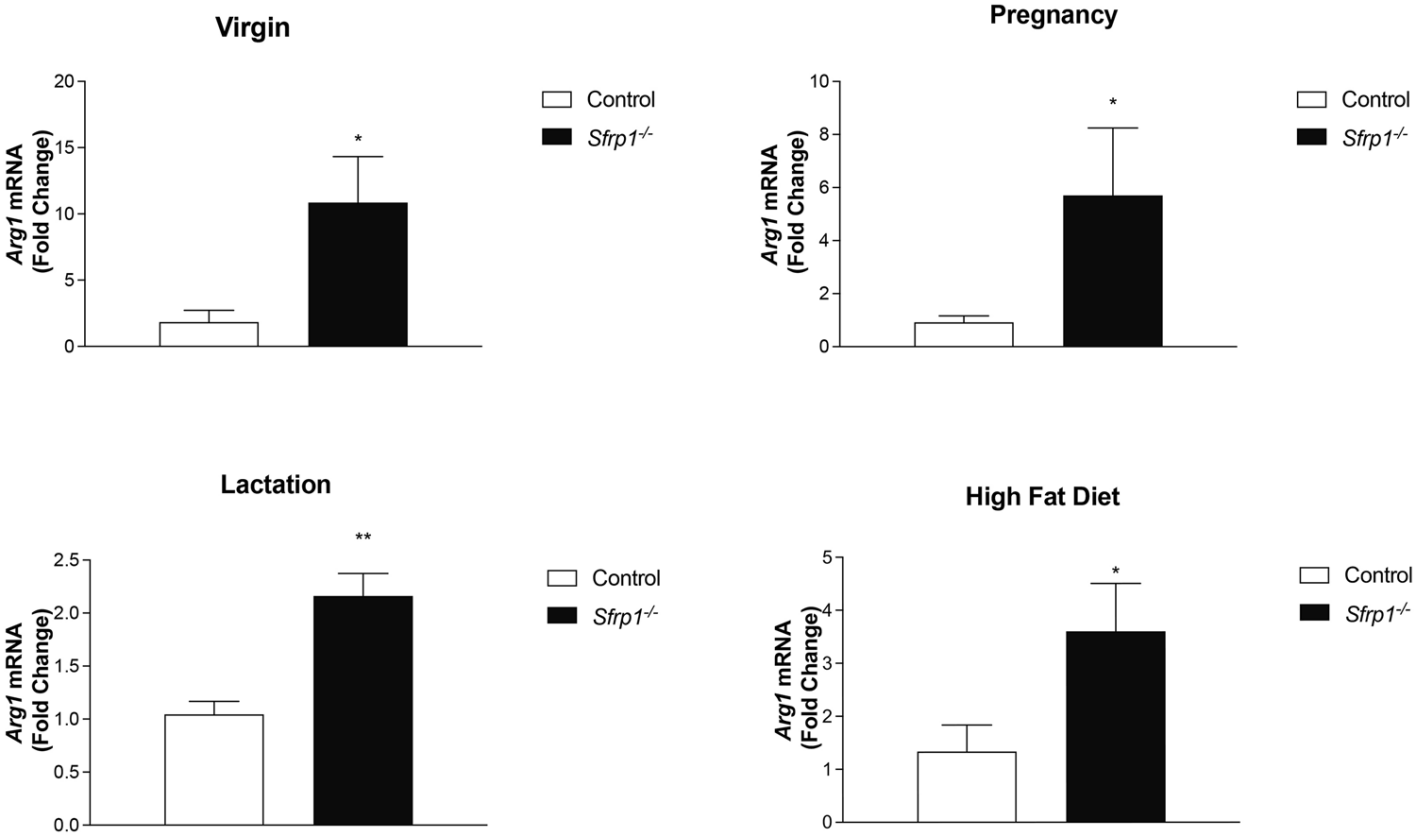
Loss of *Sfrp*1 increases the expression of a M2 macrophage marker in the murine mammary gland. RNA was harvested from the mammary glands of control and *Sfrp1*^−/−^ mice at (**A**) 10 weeks, **B** day 8 of pregnancy, **C** day 6 of lactation, and **D** virgin mice fed a HFD and mRNA levels of *Arg1* were measured by qRT-PCR. All real-time PCR results were performed in technical duplicate and results were normalized to amplification of *Actb*. Data within each bar represents *n* = 6 mice/genotype, are presented as mean ± SEM and are expressed as fold change with respect to control mice. *P < 0.05, ** P < 0.01, *** P < 0.0001 (significantly different from control mice using a Student’s *t*-test)

To investigate whether loss of *Sfrp1* affects M2 macrophage marker expression, we first looked at the mRNA expression of a murine M2 marker (*Arg1*) during different time points along mammary gland development since macrophages contribute to remodeling at all developmental stages [22–24]. We found that *Arg1* expression is significantly increased in the mammary gland from virgin, pregnant, and lactating *Sfrp*1^−/−^ mice (Fig. 1A–C). Next, we chose to look at how *Arg1* mRNA was impacted in *Sfrp*1^−/−^ mice fed a high fat diet (HFD) and our findings reveal that the expression of the *Arg1* is also significantly elevated in response to diet-induced obesity (DIO) in the mammary gland of *Sfrp*1^−/−^ mice (Fig. 1D).

### Modulating levels of Wnt3a and SFRP1 protein in benign human breast tissue impacts M2 markers

**Fig. 2:**
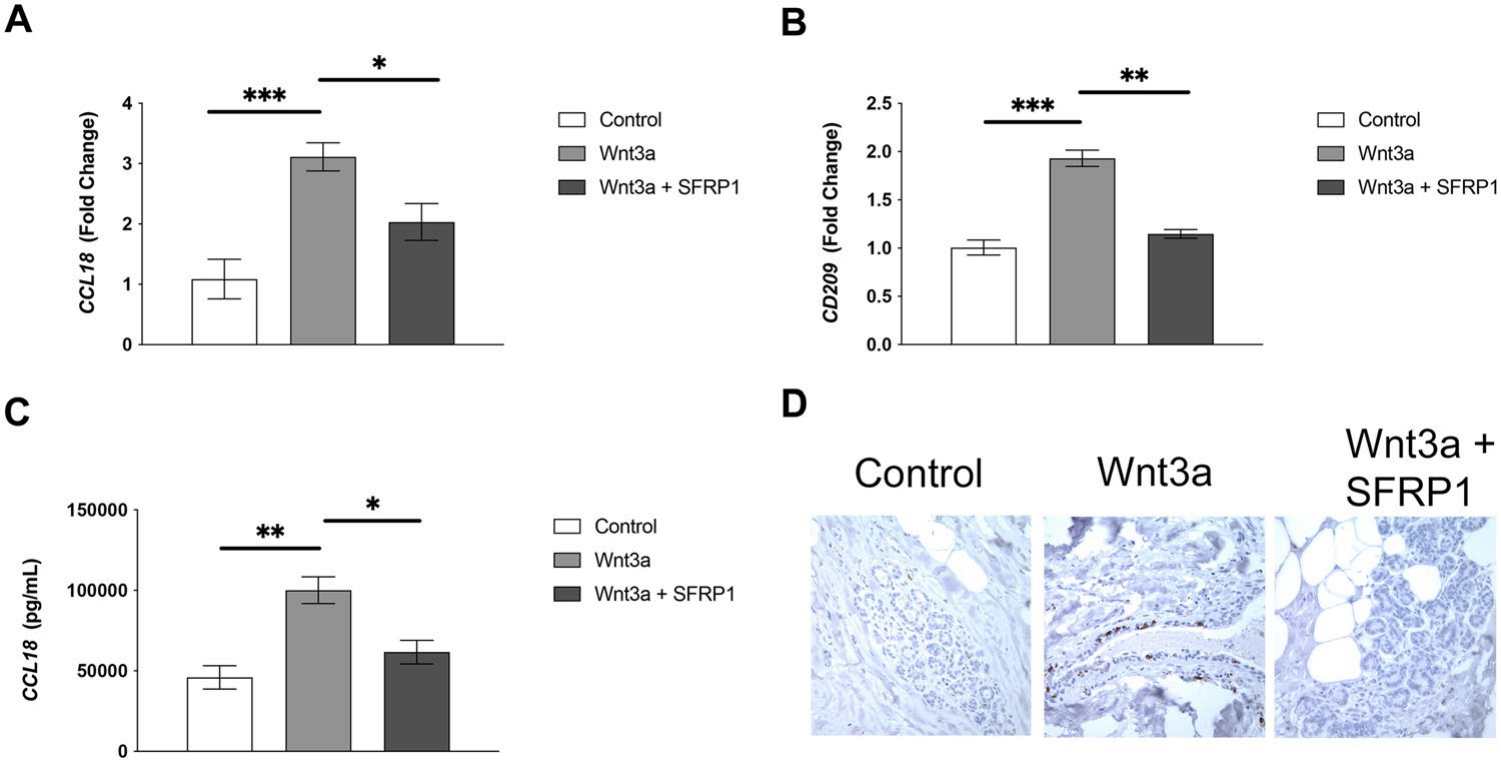
The effects of Wnt activation and antagonism on M2 macrophage polarization in human breast PDEs. RNA was harvested from Wnt3a ± SFRP1 exposed PDEs and mRNA levels of (**A**) *CD209* and **B** *CCL18* were analyzed via real-time PCR. All real-time PCR results are from technical duplicate and biological triplicate samples and results were normalized to amplification of *CD68* (macrophage marker)*.* **C** Supernatant was collected from PDEs treated with Wnt3a ± SFRP1 and CCL18 protein secretion was measured by ELISA. Bars represent mean ± SEM and are expressed as fold change with respect to control treated PDEs. *p < 0.05, **p < 0.01, ***p < 0.001 (significantly different from indicated data set using student’s *t*-test). **D** Treated PDEs were subjected to immunohistochemical analysis, stained for CD209 (brown chromogen), and representative images were captured at 100×

To examine the impact of Wnt signaling on macrophage polarization in the absence and presence of recombinant SFRP1 in a physiologically relevant environment, we employed a patient derived breast explant (PDE) system [17]. We revealed when PDEs were treated with Wnt3a for 72 h, the mRNA levels of M2 markers (*CD209* and *CCL18*) were significantly elevated in response to treatment (Fig. 2A, B). Furthermore, when Wnt signaling was antagonized by treatment with rSFRP1, *CD209* and *CCL18* mRNA expression was significantly reduced (Fig. 2A, B). Next, we wished to follow up on these data and sought to determine whether the protein levels of CCL18 and CD209 were similarly affected by Wnt signaling. Media was collected from the PDE culture and an ELISA assay confirmed that CCL18 secretion is significantly elevated in response to Wnt3a treatment and repressed when SFRP1 was added to the culture (Fig. 2C). Additionally, immunohistochemical analysis of paraffin embedded tissue confirmed that Wnt3a increased CD209 expression and that SFRP1 abrogated this effect (Fig. 2D).

### rSFRP1 can reverse a subset of Wnt-induced gene expression changes in benign human breast tissue

**Fig. 3:**
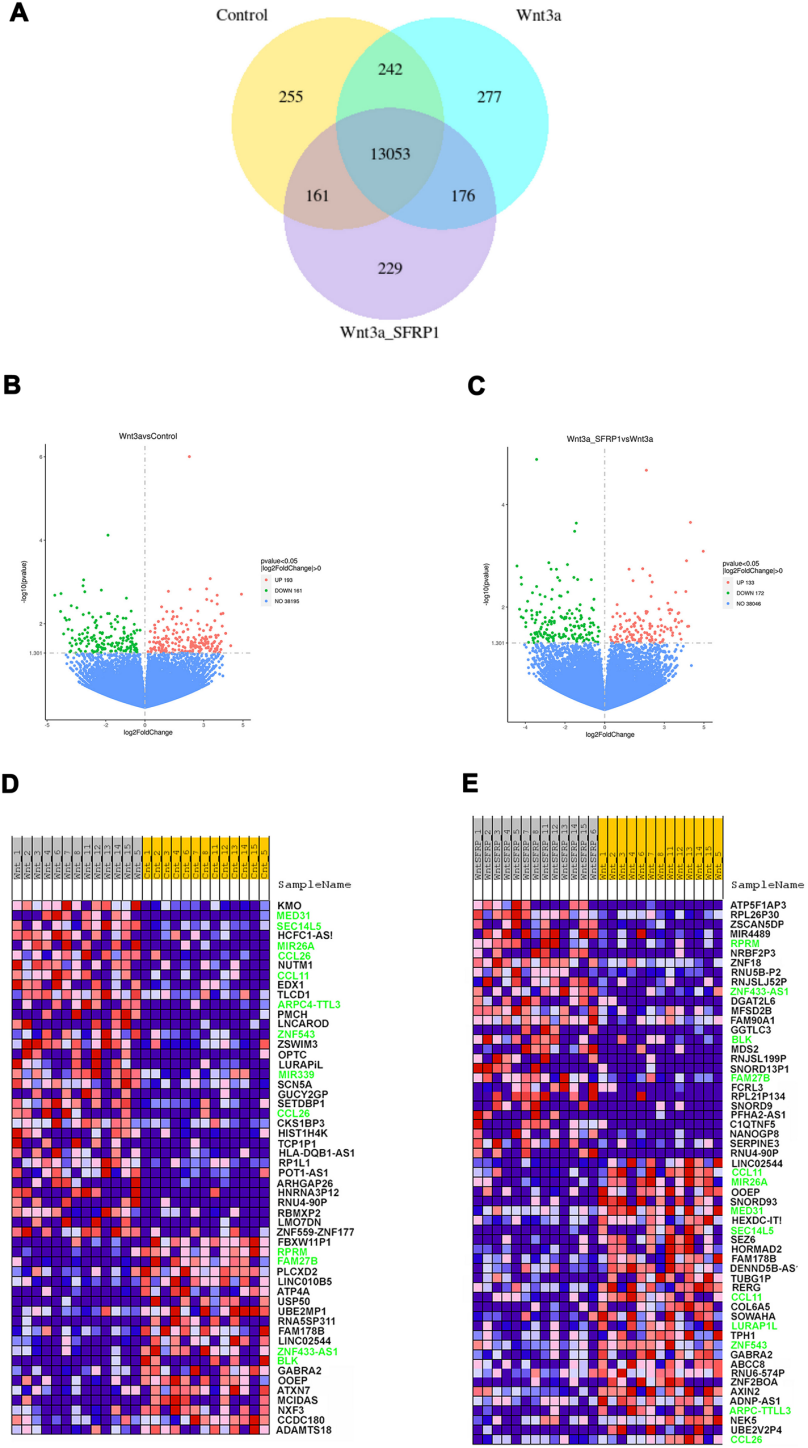
RNAseq data analysis of PDEs treated with Wnt3a ± SFRP1. Differential expression analysis of two conditions/groups (Control_Wnt3a and Wnt3a_Wnt3aSFRP1) was performed using the DESeq2R package (1.20.0). DESeq2 provides statistical routines for determining differential expression in digital gene expression data using a model based on the negative binomial distribution. The resulting P-values were adjusted using the Benjamini and Hochberg’s approach for controlling the false discovery rate. Genes with an adjusted *P*-value < = 0.05found by DESeq2 were assigned as differentially expressed. **A** Venn diagram depicting genes differentially expressed in PDEs treated with Wnt3a ± SFRP1. **B** Volcano plot of DEGs shows DEGs with FDR corrected q-values that met significance (red and green dots) appear above the FDR threshold (horizonal hashed line). Positive log_2_fold changes (red dots right of the vertical hashed line) indicate higher expression in Wnt3a compared to control treated PDEs, whereas negative log_2_fold changes (green dots left of the vertical hashed line) indicate reduced expression in Wnt3a treated PDEs compared to control (Fig. 3B). **C** Conversely, the volcano plot of DEGs in samples treated with Wnt3a + SFRP1 compared to Wnt3a samples. **D**, **E** Heatmaps of log2 counts per million (logcpm) across all the samples using the top 100 most differentially expressed (DE) genes in (**D**) Control versus Wnt3a and **E** Wnt3a versus. Wnt3a_SFRP1. Genes with an adjusted *P*-value < = 0.05found by DESeq2 were assigned as differentially expressed. Gene names colored in green represent transcripts that were both increased/decreased by Wnt3a treatment and subsequently decreased/increased by the addition of SFRP1

To gain a more agnostic view of gene expression changes in breast tissue explants in response to Wnt3a or Wnt3a plus rSFRP1, we expanded our PDE study to benign breast tissue from 13 different individuals and performed bulk RNAseq analysis. Venn diagram analysis revealed the number of genes uniquely expressed (Control, 285; Wnt3a, 277; Wnt3a_SFRP1, 229 as well as the number that overlapped between treatment groups (Control/Wnt3a, 242; Control/Wnt3a_SFRP, 161; Wnt3a/Wnt3a_SFRP1, 176 (Fig. 3A). Volcano plots provided a visual representation of the genes that were either up-regulated or down-regulated in response to Wnt3a compared to control and genes that were upregulated or downregulated in response to Wnt_SFRP1 compared to Wnt3a (Fig. 3B, E).

Genes that were significantly affected by treatment (*P* < 0.05) were used to create a heatmap and genes that were up-regulated in response to Wnt3a treatment and subsequently down-regulated in response to SFRP1 treatment are depicted in green (Fig. 3D, E). These genes include *MED31*, *SEC14L5*, *MIR26A*, *CCL11*, *ARPC4-TTL3*, *ZNF543*, *MIR339*, and *CCL26*. Genes that are down regulated in response to Wnt3a treatment and subsequently upregulated in response to SFRP1 are also shown in green (Fig. 3D, F). These genes include *RPRM*, *FAM27B, ZNF433-AS1*, and *BLK*.

**Fig. 4:**
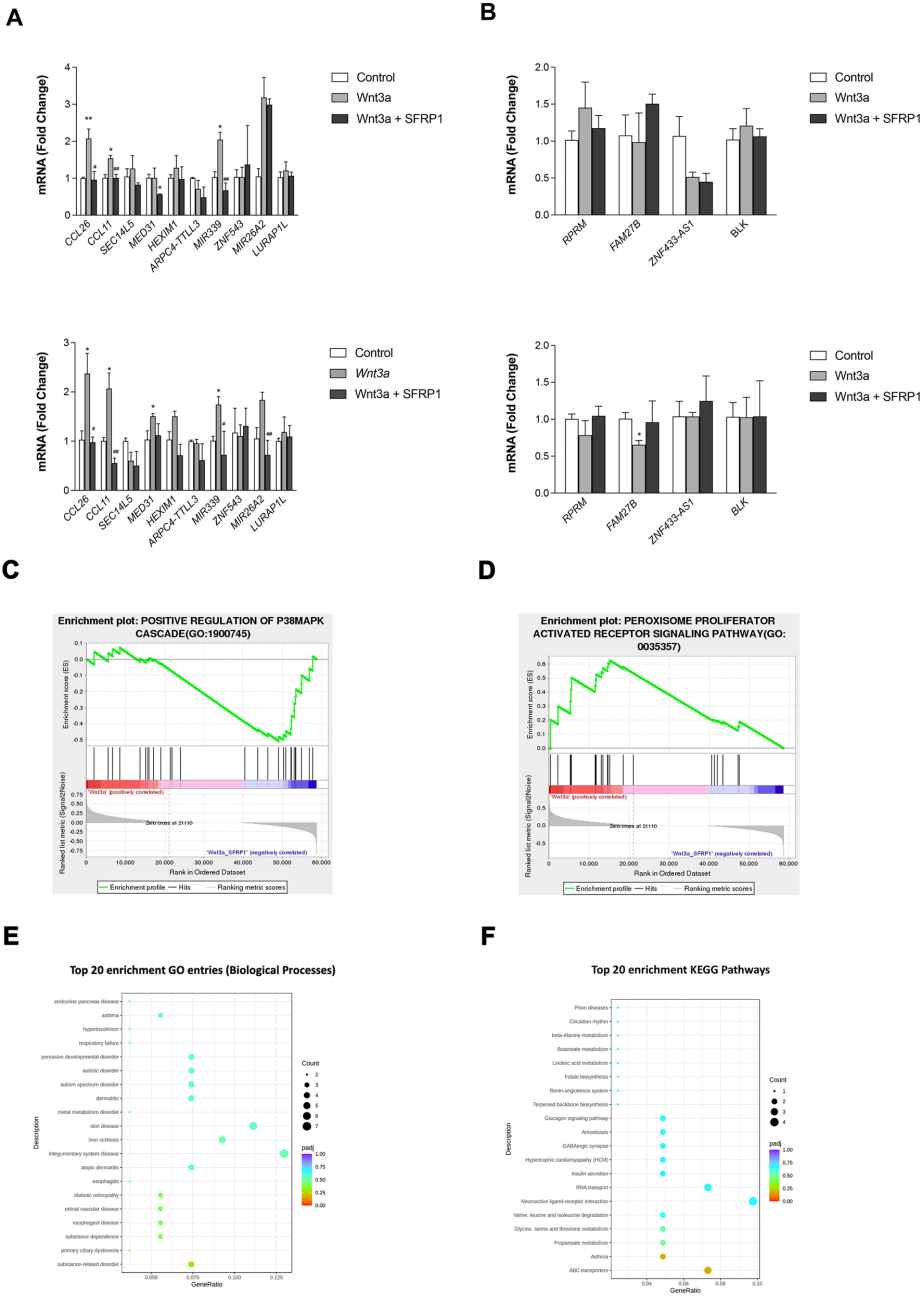
Validation of differentially expressed genes discovered by RNAseq and GSEA, GO, and KEGG enrichment analysis of the data set. RNA from 2 PDE samples in triplicate was used to confirm (**A**) the 9 genes that were upregulated in response to Wnt3a treatment that were subsequently downregulated when rSFRP1 was added using qRT-PCR. **B** qRT-PCR was also used to measure the 4 genes that Wnt3a downregulated and that rSFRP1 subsequently upregulated. Bars represent mean ± SEM and are expressed as fold change with respect to control PDEs. **C**, **D** GSEA enrichment plots show pathways that were differentially enriched in Wnt3a and Wnt3a_SFRP1 treated PDEs. **E**, **F** Top20 terms of the GO enrichment (**D**) and KEGG pathway enrichment (**E**) with differentially expressed genes *p < 0.05, **p < 0.01(significantly different from control treated PDEs using student’s *t*-test). #*p* < 0.05, ##*p* < 0.01 (significantly different from Wnt3a treated PDEs using student’s *t*-test)

To validate differential gene expression changes, we used biological triplicate RNA samples from 2 PDE patients and carried out qRT-PCR analysis (SID 1305 and 1407; Fig. 4). We confirmed that *CCL11*, *CCL26,* and *MIR339* were significantly upregulated in response to Wnt3a stimulation and the addition of SFRP1 significantly reduced their expression (Fig. 4A). To identify differences in biological processes between Wnt3a and Wnt3a_SFRP1 PDE samples we carried out gene set enrichment analysis. GESA results showed that Wnt3a treated samples were positively related to the biological phenomena involved in M2 macrophage polarization including p38 MAPK signaling (Fig. 4C) and the PPAR signaling pathway (Fig. 4D) [25, 26]. Furthermore, we performed Gene Ontology (GO) enrichment analysis on our RNAseq and many of the Top20 biological processes are inflammatory related diseases (Fig. 4E). Finally, the Top20 KEGG (kyoto encyclopedia of genes and genomes) enrichment pathways suggest that SFRP1 may be reversing metabolic changes induced by Wnt (Fig. 4E).

## Discussion

In this study we demonstrated that within a complex tissue such as the mammary gland, the targeted deletion of *Sfrp1* resulted in changes to the mammary environment resulting in more macrophages and expression of *Arg1*, a marker consistent with M2 polarization. An association between M2 related markers and Wnt3 exposure was also noted in human breast tissue. Our findings that Wnt3a drives M2 macrophage polarization in PDEs is supported by our previous findings that we can manipulate and polarize macrophages within benign human breast tissue PDEs towards M1 or M2 through the addition of IFNγ + LPS or IL-4 + IL-13 respectively [17]. Treatment with the cytokines which drive M2 polarization (IL4 + IL-13) resulted in an increased expression of CD209 and CCL18. Both CD209 and CCL18 are involved in the regulation of immune response and tissue repair, which are key functions of M2 macrophages [27, 28].

RNAseq validation revealed 3 key genes regulated by Wnt signaling and subsequent antagonism. Interestingly, MIR339 overexpression has been tied to the development of cancer by increasing cell viability and decreasing pro-apoptotic gene expression in stem cell leukemia/lymphoma cells [29]. However, we were most intrigued by our *CCL11* and *CCL26* results because both of these chemokines are involved in promoting macrophage polarization and cancer metastasis [30–32]. Tong et al. showed that high levels of CCL26 were found to be related with metastasis in differentiated thyroid cancer patients [33]. Additionally, regenerated liver phosphatase 3 (PRL-3) has been found to promote the invasion and metastasis of colorectal cancer by upregulating CCL26 to induce TAMs infiltration [32]. Levina et al. revealed that CCL11 potently stimulated proliferation and migration/invasion of ovarian carcinoma cell lines, and these effects were inhibited by neutralizing antibodies against its cognate receptors (CCR2, 3, and 5) [34]. Furthermore, CCL11 has been shown to promote lung cancer metastasis by way of Epithelial to Mesenchymal Transition, a hallmark pro-metastatic pathway [35]. Wang et al. demonstrated that the serum CCL11 level and the proportion of immunosuppressive T regulatory significantly increased in patients with breast cancer compared with healthy individuals and blockade of CCL11 in tumor bearing mice decreases immunosuppressive Tregs [36]. Tian et al. reported that CCL11 is upregulated in glioblastomas and that CCL11 promotes proliferation, migration, and invasion in glioma cancer cell lines [37]. Future work will be required to study the effects of CCL11 and CCL26 on M2 macrophage polarization and the subsequent effect on the migration and invasion of breast cancer cells. Our GSEA data unveiled that signaling pathways affected by Wnt stimulation/antagonism are involved in M2 macrophage polarization. Specifically, p38MAPK signaling is required for polarization in macrophages derived from murine bone marrow and PPAR signaling initiates the development of M2 macrophages by promoting the expression and ligation of *α*_v_*β*_5_ integrins [25, 26]. Finally, our GO and KEGG enrichment data provide an exciting avenue to pursue going forward in terms of the effects of Wnt antagonism in breast tissue.

In conclusion, our data demonstrates that exposure of human benign breast tissue to Wnt is associated with changes in levels of genes and pathways affiliated macrophage polarization an global inflammation. These findings suggest that SFRP1 may be critical target to develop as a therapeutic strategy to help disrupt the tumor microenvironment.

### Supplementary Information

Below is the link to the electronic supplementary material.

Supplementary file1 (DOCX 16 KB)

## Data Availability

The datasets generated during and/or analyzed during the current study are available from the corresponding author on reasonable request.
