# Supplementary material for: SFRP1 decreases WNT-Mediated M2 macrophage marker expression in breast tissue
Source: Cancer Immunol Immunother. 2024 Mar 30;73(5):86. doi: 10.1007/s00262-024-03638-0 (PMC10981600; doi:10.1007/s00262-024-03638-0)
Supplement: Supplementary file 1 — Supplementary file1 (DOCX 16 KB) [file 262_2024_3638_MOESM1_ESM.docx]

| **Amplicon** | **Forward Primer** | **Reverse Primer** |
| --- | --- | --- |
| *Arg1* | 5'-AGG AAC TGG CTG AAG TGG TTA-3' | 5'-GAT GAG AAA GGA AAG TGG TGT-3' |
| *CCL26* | 5'-GCA CTT TGA GAG GCC GAG AA-3' | 5'-TGA GAC GGC CCC TCC AG-3' |
| *CCL11* | 5'-TCC AAC ATG AAG GTC TCC GC-3' | 5'-GTG GTT GGG ACA GAA GCT GG-3' |
| *SEC14L5* | 5'-CCT TCC ACA GCT GTC CTG G-3' | 5'-GGT GTG CAC AGA GCC TGA-3' |
| *MED31* | 5'-GGT GGC GTT TTG GTG TCT TC-3' | 5'-AGT CGA TTT CCA GCA TCA TCT GT-3' |
| *HEXIM1* | 5'-TTT ATT GGG GTG CTC CGC TT-3' | 5'-GCA ATC TGG GGA GCT CAA GT-3' |
| *ARPC4-TTLL3* | 5'-GAT GGT ACC GTC CGG AAG TC-3' | 5'-GGA GAG TGG CAG TCA TCG C-3' |
| *MIR339* | 5'-CAG ACA CTG GGG CAG GC-3' | 5'-ACC TGC GGA CTT CTC ATG TC-3' |
| *ZNF543* | 5'-ACC TGC GGA CTT CTC ATG TC-3' | 5'-GGG TTT TGT GTT GTC ACC TGG-3' |
| *MIR26A2* | 5'-GCT GTG GCT GGA TTC AAG TA-3' | 5'-AGC TGC CTC CAG AAA CAA GTA A-3' |
| *LURAP1L* | 5'-TGG AGA AGC TGG AAA GTG GAA-3' | 5'-TAC GGT ATC AGG CAT CCC CT-3' |
| *RPRM* | 5'-CTG CGA ATT TGA ACG GGG TG-3' | 5'-GCA GTC ACG AGC TAC GAG TT-3' |
| *FAM27B* | 5'-GGG AAT GGG AGA TGG CAA CA-3' | 5'-GTG GGG TTC CTA AAG GGT GG-3' |
| *ZNF433AS1* | 5'-CCC TCC TCC GTA GCT TTT GT-3' | 5'-AGC TTC CAG ATA TTC CGG CG-3' |
| *BLK* | 5'-TGG TGT TGG AAG TTG CTC GT-3' | 5'-CTT ACC AGC CCC ATC CTT GG-3' |

**Table 1.** Primers utilized for real-time PCR analysis
